# Experiences and recommendations from people with spinal cord injury following participation in a disability education session at an allopathic medical school: a qualitative study

**DOI:** 10.1038/s41394-023-00582-6

**Published:** 2023-07-07

**Authors:** Renée Pekmezaris, Vidhi Patel, Paige Herman, Adam B. Stein, Ona Bloom

**Affiliations:** 1 Division of Health Services Research, Dept. of Medicine, Institute of Health System Science; Donald and Barbara Zucker School of Medicine at Hofstra/Northwell, Manhasset, NY USA; 2 Department of Medicine, Massachusetts General Hospital; Harvard Medical School, Boston, MA USA; 3 Department of Physical Medicine and Rehabilitation, Donald and Barbara Zucker School of Medicine at Hofstra/Northwell, Manhasset, NY USA; 4 The Feinstein Institutes of Medical Research, Northwell Health, Manhasset, NY USA; 5 Department of Molecular Medicine, Donald and Barbara Zucker School of Medicine at Hofstra/Northwell, Manhasset, NY USA

**Keywords:** Health care, Health occupations

## Abstract

**Background:**

Students in half of US medical schools do not receive formal instruction in providing medical care for people with disabilities. To address this gap in training, our medical school developed several strategies, including a session for second year medical students to address communication skills, knowledge, and attitudes relevant to delivering healthcare for people with disabilities. Here, our objective was to explore perceptions of people with spinal cord injury (SCI) who participated in the session on its content and structure.

**Methods:**

Qualitative research using a focus group of people with SCI who participated in an educational session for medical students in an LCME accredited allopathic US medical school. A purposive sample of adults with SCI (*N* = 8) participated in a focus group. Data were analyzed using a six-phase thematic analysis.

**Results:**

Participants favorably viewed the educational session, felt their participation was valuable, and had suggestions for its improvement. Four major themes were identified: (1) session format, content; (2) addressing student discomfort and avoidance behaviors; (3) increasing student knowledge and preparation; and (4): important lessons from discussions of past and role-played doctor-patient interactions.

**Conclusions:**

First-person input from people with SCI is critical to improve medical education and healthcare provision to the SCI community. To our knowledge, this is the first study to report feedback from stakeholders providing specific recommendations for teaching disabilities awareness to undergraduate medical students. We expect these recommendations to be relevant to the SCI and medical education communities in improving healthcare for people with SCI and other disabilities.

## Introduction

Persons with disabilities (PWDs) comprise the largest minority group in the US, with approximately 20% of Americans reporting difficultly performing a major life activity [1]. PWDs experience effects of widespread health disparities, especially with regard to preventative care and health maintenance [2–4]. PWDs report less satisfaction with medical care they receive than those without disabilities and they report difficulties in accessing care due to their disability [2, 3]. Reasons for these disparities are complex, including: (1) physical accessibility impediments, (2) communication barriers, (3) financial challenges, (4) health literacy issues, (5) and others [2]. While PWDs utilize healthcare more frequently than persons without disabilities, only 50-52% of medical schools report disability awareness education [2, 4, 5].

Perhaps the group of PWDs most at risk of experiencing health disparities in the US are the ~350,000 people living with traumatic spinal cord injury (SCI) [6, 7]. Due partly to reduced mobility, people with SCI are at significant risk for developing medical conditions including: (1) obesity, (2) type II diabetes mellitus, (3) respiratory infections, (4) muscle atrophy, (5) pressure ulcers, (6) kidney stones, (7) venous thrombosis, (8) osteoporosis, (9) atherogenesis, (10) and other medical consequences that increase risks of stroke and coronary heart disease [6, 7]. Impaired mobility also often has psychosocial effects that negatively impact quality of life, including: (1) social isolation, (2) diminished social participation, (3) and more frequent anxiety and depression [8, 9]. Given these challenges for people with SCI, there is a critical need to improve medical education on this topic and to include all stakeholders in this process.

Federal laws such as Section 504 of the Rehabilitation Act and Americans with Disabilities Act (ADA), prohibit discrimination in inpatient and outpatient healthcare settings. Despite this, people with SCI often face barriers to accessing medical care and to participating in clinical trials [10, 11]. Barriers to healthcare for PWDs have been highlighted by the ongoing COVID-19 pandemic: a report issued in April 2020 from the United Nations Human Rights Office of the High Commissioner acknowledged that PWDs “are disproportionately impacted due to attitudinal, environmental and institutional barriers that are reproduced in the COVID-19 response” [12].

To enhance medical care for people with SCI and other disabilities, it is critical that medical school curricula address the topic of providing medical care for people living with physical challenges [10]. Medical schools that do offer instruction on this topic use varied approaches including: (1) lectures, (2) hands-on exercises to simulate the experience of living with disability, (3) shadowing physiatrists and other professionals who serve people with SCI or other disabilities, (4) in-home encounters with PWDs, (5) and standardized patient interactions [10, 11, 13, 14]. Researchers have shown that sessions specifically focused on preparing medical students to provide care for people with disabilities improve their understanding of disability and the barriers faced by PWDs in obtaining healthcare [13].

**Fig. 1:**
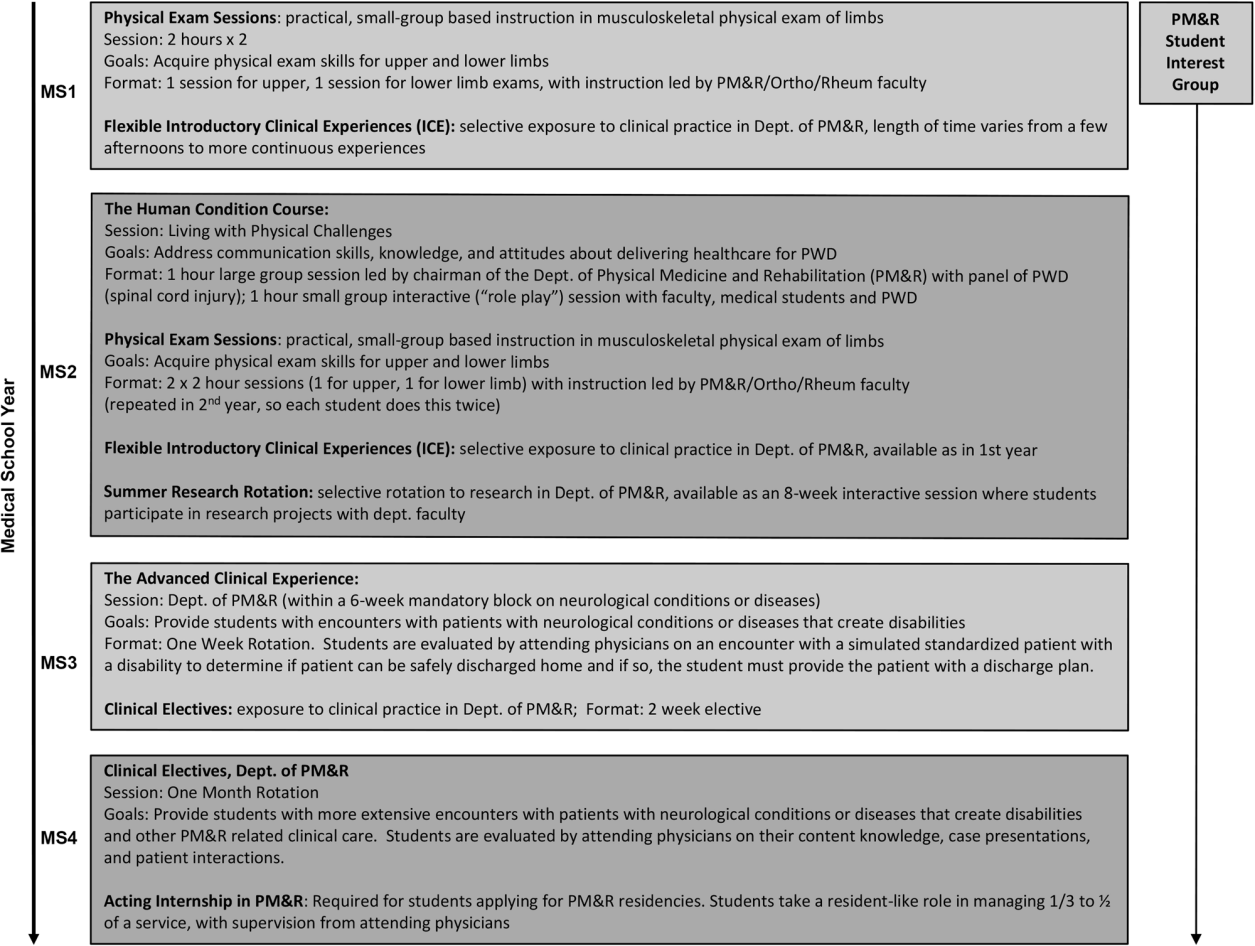
Educational Activities Related to Providing Healthcare for People with Disabilities. For each medical school year (MS) 1–4, educational activities related to providing healthcare for people with disabilities are listed and described.

Faculty at our medical school have tried to address education on barriers faced by PWD in obtaining healthcare in multiple ways (Fig. 1). First, they developed a two-hour course for second-year medical students to address communication skills, knowledge, and attitudes pertaining to delivering healthcare for people with disabilities, including people with SCI [15–21]. Developed for the neuroscience course, entitled “*The Human Condition*,” this annual session is held during the spinal cord curriculum module, after review of neuroanatomy, clinical presentation, pathology, and basic science related to SCI [14].

The session begins with a lecture from the chairperson of the Dept. of PM&R to present the conditions and diseases typically treated by physiatrists. This is followed by brief presentations from community members with SCI who share some of their lived experiences. Next, the medical students attend interactive small group session in which they interact directly with a person with SCI to role play an initial wellness visit (“check up”) with a physician. The small group also includes a medical school faculty member who is trained in medical communication skills and a faculty member from the Dept. of PM&R who serves as a content expert. Many of the persons with SCI have served in this role for multiple years. From 2013-2022, the course was attended by approximately 880 medical students.

The PM&R curriculum continues into the third year of medical school, when all students build on the experience and knowledge gained in this session during a one-week rotation in the Department of PM&R, which is focused on encounters with patients with neurologic conditions or diseases that create disabilities. After that experience, students are evaluated on an encounter with a simulated standardized patient with a disability, on whom they must perform a functional assessment and use their findings to determine if the patient is able to be safely discharged home and what their discharge plan should include. In addition, throughout their four year education, medical students at our institution can enrich their knowledge of issues related to providing healthcare to PWD by participating in a PM&R student interest group, where they discuss topics and hold educational sessions related to the practice of PM&R, including treating patients with SCI and other disabilities (Fig. 1).

In January 2020, the second year educational session was held in person. After the session, a focus group of people with SCI who participated was held to garner their feedback. Here, the goal of this study is to describe the perceptions of persons with SCI who participated in the session and report their priorities and recommendations for its improvement.

## Methods

### Didactic session content and format

A full description of the course has been presented previously [13]. Briefly, prior to the session, faculty who participated in the course were provided with: (1) a guide that included the goals and learning objectives of the session, (2) required reading for the students, (3) an overview of the schedule containing both a large group format and small group components, (4) the role of the faculty members in the small groups, (5) and suggested questions and prompts for the small groups. The goals of the course are for students to: (1) gain an appreciation of the perspectives of people living with physical disabilities, (2) develop skills for eliciting histories and performing physical exams with patients with physical disabilities, (3) and to develop an awareness for the field of PM&R [14].

Learning objectives for students are: (1) to be able to define disability, (2) define major challenges to obtaining healthcare among people who are disabled, (3) describe how disability rates vary by social determinants of health such as age, sex, race, ethnicity and socioeconomic status, (4) describe roles that major pieces of legislation have had on the ability of people who are disabled to maintain their health, (5) identify some common barriers encountered by people with disabilities to obtaining healthcare, (6) compare and contrast activities of daily living (ADLs) and instrumental activities of daily living (IADLs), (7) and to describe best clinical practices for obtaining a medical history and physical exam for patients with physical disabilities. The small group session discussion prompts were: (1) Student experiences with patients with physical challenges, (2) experiences of SCI panelists (best/worst encounters) with physicians and the health care system, (3) introductions to an initial intake and role playing of a patient encounter that includes a physical exam, (4) role playing of obtaining a medical history that includes a review of ADLs and iADLs, prevention history, social history, and review of systems, (5) discussion of performing a physical exam that includes consideration of environmental (exam room) barriers, and (6) planning for any necessary follow up care. In preparation, students were asked to read: (1) a textbook chapter on physiatric history [22], (2) a message from the Surgeon General on equity for PWDs [23], and (3) a New York Times perspectives article on the disabilities rights movement [24]. This last item in the reading list is updated yearly to reflect a current article related to the disabilities rights movement or other topic related to healthcare of PWD.

The course begins with all students receiving a brief framing lecture who is a physiatrist board-certified in SCI medicine, followed by presentations from a panel of three persons with SCI who share their lived experiences. This is followed by a 70-minute, small group interactive session led by a medical school faculty member with expertise in communications and attended by a Dept. of PM&R faculty member, medical students and an individual with SCI. Each person with SCI is assigned to one small group of 8-12 students, where the person shares their lived experiences, and play the role of a patient who is presenting to a primary care physician for an initial encounter, to elucidate teaching points to the medical students. The small group discussion includes students reflecting on prior interactions with PWDs, after which persons with SCI describe their prior encounters (good and bad) with healthcare professionals. In a continuous “role play” format, medical students perform a first encounter medical exam and history with the person with SCI, with students in the small group participating sequentially. Faculty and patients highlight points for consideration, such as what to include or modify during a physical exam, wheelchair accommodations, and when to interview patients with or without caregivers. The session ends by reviewing key points and distributing a brief internal educational resource guide prepared by our faculty for students on best practices for conducting an initial intake with a person with physical challenges, as well as definitions of ADLs and iADLs.

### Focus group participants, data collection, and analysis

This is a qualitative study of a purposive sample of adults with chronic SCI who are wheelchair users for community mobility (Tables 1,2) and were recruited from the local area to participate in the educational session [14]. The local Institutional Review Board (IRB) deemed this study exempt from IRB review. All participants provided permission to be recorded during the focus group for the purposes of transcript analysis. Following the 2020 session, all participants with SCI were invited to attend an in-person, 1.5-hour focus group to provide feedback on the session and to identify opportunities for its improvement. (The session was held prior to the declaration of the COVID-19 pandemic in the US.) Eight participants with SCI agreed to attend (Table 1); reasons for declining to attend were not recorded. The focus group was moderated by two investigators (RP, VP) with qualitative research experience. Investigators (RP, VP) used a moderator guide with open-ended questions and probes related to topics including lived experiences and priorities for teaching about relevant medical care (Appendix 1). The discussion was digitally recorded, stored on an internal password-protected server and transcribed professionally. Transcripts were checked against the original recording for accuracy.

**Table 1:** Clinical and demographic characteristics of focus group participants.

	Chronic SCI
**Participants (N)**	8
**Males N, %**	6, 75%
**Age, years (**Mean ± SEM**) Range**	48 ± 14.5 27-71
**AIS grade, N (%)**	
A	6 (75%)
B	1 (12.5%)
C	1 (12.5%)
**Level of Injury, N (%)**	
Cervical	6 (75%)
Thoracic	2 (25%)
**Wheelchair user**	
Power or power assist	7 (87.5%)
Manual	1 (12.5%)

**Table 2:** Participant quotes from the focus group session.

Theme	Thematic quotes from focus group participants
**Theme 1: Session Format, Content** Subtheme 1: Session Length	Quote 1: “I agree. I think it’s too short. They-after-after twenty or twenty-five minutes everybody’s getting on the groove; for the first person.” Quote 2: “That was towards the end, so it was, again, with a time constraint. It was a little rushed.” Quote 3: “Honestly, I think to do it right-really do it right and get the most out of it-it’s too short.”
Subtheme 2: Session Format	Quote 1: “So maybe you can put them on a timer, you know? Give them a real… No, give them a real… Like, if you are saying there should be more time for this and everything, figure out how many students you have and just say, ‘Okay. You have seven minutes.’” Quote 2: “Perhaps it would be helpful if they had a list of questions that they wanted to ask us ahead of time. Even if they were anonymous questions, so they don’t have to be that person who had to ask that question; do you know?” Quote 3: “And as much as you want them to be thinking on their feet and be spontaneous, it may be beneficial for them to be given a loose script ahead of time.”
Subtheme 2: Advanced Preparation by Students	Quote 1: “So, I think they-if they were a little more prepared, knowing, “Okay. You are going to do a role-playing where you’re going to have a doctor’s visit.” I don’t know how many of them knew that in advance.” Quote 2: “And maybe the night before they could tell them to: ‘This is what we are doing. Be prepared to be asking as if you were the doctor and you are going to have seven minutes.’ So, you know.” Quote 3: “Vocabulary. Maybe you should give them a list of vocabulary that’s common for us: incomplete, complete.”
**Theme 2: Addressing Student Discomfort and Avoidance Behaviors**	Quote 1: Hm-I used the elephant in the room. I also told them, ‘Is there anything that you have in your head that you are thinking or want to ask me that I haven’t been asked before? So, just ask it. Let’s get it over with.’” Quote 2: “They were all hesitant at first in my group. I had to-and I’m used to this-like, call people out and it makes them uncomfortable at first, but then it makes them more comfortable.” Quote 3: “I think that, also, probably having a professor encourage the students and say, ‘There are no wrong answers; there are no wrong questions.’” Quote 4: “Yes. It’s all about the learning and not really, ‘Don’t feel awkward,’ you know-things that like that.’”
**Theme 3: Increasing student knowledge and preparation**	Quote 1: “Whether it’s an eating cup or, you know, if we are driving or whatever modifications we need to do anything. So, a list of that stuff would be helpful.” Quote 2: “’Everybody’s different.’ I said, ‘For me-for you-you get up out of bed, you put your pants on, you go get a cup of coffee, brush your teeth, and you don’t even think about it like we do.’ I said, ‘That’s a two-hour operation for me.’” Quote 3: “’I said, ‘I take a shower and I get dressed and you have to understand something: I’ve just had a big athletic event.’’”
**Theme 4: Important lessons from discussions of past and role-played doctor-patient interactions**	On direct communication with patients: Quote 1: “They don’t have confidentiality, so maybe the way to address it is, you know, ‘You are here today as my patient.’ You know, ‘Will this person be participating in it? Would you like, you know, him to stay?’ Because sometimes I think people might feel uncomfortable saying, ‘No. I don’t want you here. Can you step outside?’ Because they are our arms, our legs, our caregivers. So, you don’t want to say, ‘I don’t need you here for this,’ but, then again, you are your own person. So, I suggested, you know, possibly using the Hippocratic Oath and say, ‘I can’t talk to [him/her] unless you tell me it’s okay.’” Quote 2: “I said, ‘So, when you talk, when you become doctors and they come in with a caregiver, a sister, a brother, or a wife, whatever, don’t be talking. Talk to the patient.” On unpreparedness of past physicians in initial encounters: Quote 1: “I said, “Did you not read the chart? I’m paralyzed. Do you know what that means?” So, I think looking at the situation, you know, something like that is something they need to be more aware of.” Quote 2: “I had a work doctor ask me, ‘Why can’t you get up anymore?’. I said it was because of my injury and he said ‘Well, let’s see you try.’” Quote 3: “I had a doctor ask me if I was-if I had cerebral palsy.”

With the goal of optimizing credibility, transferability, and dependability of results, we utilized constant comparison, and kept a record of decisions made during the analysis. The transcript was analyzed by three investigators (PH, OB, VP), to gain a complex understanding of the data. A six-phase thematic analysis approach was utilized [25]. In phase one, transcripts were reviewed multiple times independently to become familiar with the data. Researchers documented initial thoughts, potential codes and themes. In phase two, researchers focused on patterns among their potential codes and themes and then used inductive and deductive coding to generate a comprehensive code set. Two researchers (PH, OB) together documented their rationale for coding transcript text and explained to the other researchers how the data were perceived and examined. Phase three included searching for common themes and collating codes. In phase four, themes were reviewed and refined by a third researcher (VP). Criteria for retaining themes were that they needed to be specific and concrete, but broad enough to capture ideas. Themes with sparse data were eliminated and themes with more data were subdivided. In phase five, team members (PH, OB, VP, RP) met and discussed finalization of theme names. In phase six, the report was generated and discussed with all investigators.

## Results

Individuals who participated in the focus group were people with SCI (*N* = 8: 3 males, 5 females), who were wheelchair users for community mobility (Table 1, which includes a referral to the ASIA/ISCoS International Standards for the Neurological Classification of Spinal Cord Injury) [26] and who volunteered to participate in the session. The discussion explored their perceptions, thoughts and ideas, including recommendations to improve student communication, student knowledge levels and familiarity with PWDs, approaches to familiarize students with challenges of disability, utility of role playing, and student comfort in asking about ADLs and IADLs. Four major discussion themes with subthemes emerged from a thematic analysis of the focus group transcript. Table 2 provides quotes from focus group participants that support each theme.

### Theme one: session format and content

Several focus group participants reported that the role play portion of the small group session was too brief. They also recommended adding a second session to be held later in medical school training, when students could practice with more advanced communication skills, such as better eye contact with patients. (See Table 2 for participant quotes.) If this was not possible, then they suggested adding time (~15 min) to role playing, even at the expense of the large group lecture. It was suggested that students be directed to ask about patient goals for the visit. Another participant suggested that students prepare specific questions for participants prior to the session, or use the format of a pre-written script. Each of these suggestions were supported by the group. While the role play was enthusiastically embraced by focus group participants, suggestions were made to include different scenarios, e.g.: variations in disability type, patient demeanors, and functional abilities. A suggestion was made to time role playing, to facilitate more equal participation among students. It was also recommended that students be asked by faculty to pose questions specifically about physical activity and sexual activity. Subtheme 1: Session Length. Several participants noted that session was too short to cover all of the topics that they felt were important to convey to students. Subtheme 2: Session Format. There was significant discussion among focus group participants regarding representation of all disability types. Participants also discussed the importance of balancing role play interactions to be realistic, as well as educational, highlighting that some patients may be less friendly and open than volunteers donating their time to promote medical education.

### Theme 2: addressing student discomfort and avoidance behaviors

Participants perceived some hesitancy and awkwardness among medical students who were asked to initiate or continue patient interactions, specifically around aspects of disability. One participant commented that this younger generation of learners was raised in a world where in general, live communication is decreased and electronic communication is increased. (Note that the session was held prior to the COVID-19 pandemic, during which use of electronic communication has expanded.) Participants with SCI suggested that students should be asked to role play diagnosing a standard clinical vignette, such as a urinary tract infection (UTI), and that participants with SCI prompt students to ask them questions about sex. Also, the importance of looking the patient in the eye was emphasized.

### Theme 3: increasing student knowledge of ADLs and Preparation

#### Subtheme 1: advanced preparation by students

While advanced preparation was required of students, several focus group participants discussed possible improvements in student preparation. Specifically, they recommended: (1) prior submission of questions to patients from students; (2) informing students that they are expected to ask about specific topics, including physical activity, and patient visit goals, (3) potential use of a script with questions for patients; and (3) a greater understanding of the range of functional abilities experienced by PWDs.

#### Subtheme 2: knowledge of ADLs

A related but distinct theme was that participants were surprised at the limited knowledge of 2nd year medical students on specific topics. They expected deeper medical knowledge and familiarity with common terminology, such as ADLs, IADLs, and physician visits, so that they could address these topics in patient interviews. They were also surprised that some medical students were unaware of: (1) needs for adaptations to perform ADLs, (2) how environmental conditions like temperature may influence a person’s dexterity/hand function, or (3) how abnormal pain or sensory perceptions may influence symptom reporting. In this context, participants also discussed the importance of when to perform a complete physical exam. For example, it may not be necessary in each patient encounter to ask someone with SCI to remove their clothing in order to don a medical gown. However, there may be other occasions when asking someone if they are experiencing symptoms may be inadequate, such as when someone with SCI experiences a fall and may be unaware of a foot fracture, due to their lack of sensation.

### Theme 4: important lessons about doctor-patient interactions

Much of the focus group discussion centered on aspects of the doctor-patient interaction that participants thought were important for medical students to learn. The first lesson aimed to teach a medical student the importance of knowing how to ask a caregiver to leave the room and how to talk to/examine the patient alone, because (as with any patient) there may be unfavorable power dynamics, privacy issues, or even caregiver abuse (Table 2). In some cases, the patient may fear reprisal from the caregiver if they request privacy. A related lesson was that a clinician should communicate directly with the patient and not through a caregiver, emphasizing autonomy. A third lesson shared by participants was the impact of abnormal sensation when reporting healthcare problems. For example, people with SCI are at increased risk for bone fractures due to accelerated osteoporosis and may have other injuries for which they do not have complete physical awareness, such as pressure ulcers. Alternatively, students should appreciate that not every medical issue or patient visit goal requires a patient to don a gown, which can be laborious and time consuming. Although the original session guide suggested that the role play be conducted as an initial patient encounter in a continuous manner, where multiple students participated in taking different parts of the patient’s medical history, not all small groups made the same interpretation. Thus, a fourth important lesson was to perform a thorough review of the medical chart prior to meeting a new patient. This would facilitate expectations of the patient’s functional status prior to initiating the exam, avoiding inappropriate requests and tailoring the history and physical. An example was given of a patient who was asked by a physician to stand during an exam, although they were physically unable to do so. Finally, participants stressed that generally, wheelchair users prefer not to be treated differently than others.

## Discussion

Many studies have explored communication skills of medical students (e.g. [27]). Educational standards for medical education address competencies regarding care of people with disabilities, including the Liaison Committee on Medical Education that requires for accreditation: *“clinical instruction must include … the important aspects of preventive, acute, chronic, continuing, rehabilitative, and end-of-life care…”* Despite this, many students begin and complete medical school with little or no exposure to people with SCI or other disabilities [28]. A recent online survey reported data from 75 accredited allopathic and osteopathic American medical schools on the presence of a disability awareness program [4]. While 52% of schools reported having a designated program to instruct medical students on specific challenges of PWDs related to their healthcare, the most common format was PWDs or caregivers speaking in a large group setting [4]. This educational gap on the challenges of PWDs related to their healthcare, renders early career medical doctors unprepared to optimally address the needs of patients with SCI or with any other physical, intellectual, or emotional disabilities that they are likely to encounter in clinical practice, and/or to fully understand ADA compliance [21]. The rationale for including persons specifically with SCI in this educational session was that, due to reduced mobility and sensory impairments, as well as autonomic dysfunction, they are at highly increased risk of multiple medical consequences of SCI, and wheelchair users often experience the most severe healthcare access issues. In parallel with the growing call to include people with SCI in partnership with researchers in designing studies, there should be an increased emphasis on including people with SCI or other PWD in medical education.

There are many reasons why first-person input from people with SCI and other disabilities is critical for the process of improving medical education [29]. The first reason is authenticity. The performance of a standardized patient without a disability in role playing may lack nuanced understanding that only a person with a disability can provide [21]. A related reason is patient-centeredness [10, 13]. Attempting to improve curriculum without direct patient input may perpetuate misconceptions, ultimately adversely influencing student knowledge and performance. For example, our focus group discussion demonstrated that clinicians may “miss” important issues because the patient with a disability may not be able to sense pain and may therefore not bring a pain-related issue to a clinician’s attention.

Interestingly, perhaps the strongest recommendation from focus group participants was to increase the length, frequency, and student participation in the continuous role play, consistent with prior literature [21, 30]. While the utility of role play is not new, the present study provides novel *specific* recommendations: repetition of a similar session later in undergraduate medical education, script provision before the session to facilitate student participation and learning, allotting more time for repetitive skills building, the importance of querying ADLs and IADLs, inclusion of patients with varied disabilities, and role playing with varied patient demeanors. Participants also suggested prompting learners to ask about sexuality and to directly query patient visit goals. There were other patient-oriented recommendations, such as prompting medical students to “look the patient in the eye”, directly addressing a patient’s disability, and using a standardized clinical vignette, such as a urinary tract infection. Recommendations from focus group participants to medical students, such as asking a caregiver to leave the exam room to facilitate trust/confidentiality, as well as to facilitate potential reporting of caregiver abuse, are not specific to people with disabilities.

### Limitations

This qualitative study was based on data from a small group of people with SCI who participated in a disability education curriculum of a medical school in the NY metropolitan area. This group of people with SCI does not reflect national demographic characteristics, where males comprise 80% of the population [7, 31]. The scope of this analysis is limited to this focus group experience and is not intended to describe the entire multi-year curriculum aimed at increasing disability education. Participants may not be representative of other patient groups, in terms of age, type of disability, cultural background, emotional demeanor, or other clinical and demographic variables.

## Conclusions

While it is impossible to prepare medical students for every potential scenario, 20% of Americans report difficultly performing a major life activity [1]. With increased longevity, the proportion of Americans who experience a disability will likely increase. Thus, it is critical that medical students are prepared with the skills and knowledge required to provide excellent medical care for people with disabilities. Here, we present our experience on meeting this goal with input from people with SCI. People with SCI recommended changes in session format and content, as well as suggested ways for students to arrive better prepared for the session, and specific areas of student knowledge that they felt should be increased. They also provided examples of aspects of the doctor-patient interaction that they perceived as important for medical students to know, in order to provide optimal medical care to people with disabilities. Additional studies are needed to explore results of implementing recommendations from focus group participants, if knowledge translation occurred as a result of this educational session and if it resulted in improved medical care for PWD.

## Supplementary information

Appendix 1

AJ Checklist

## Data Availability

Data from the current study may be made available from the corresponding author upon reasonable request.
