# Supplementary material for: Experiences and recommendations from people with spinal cord injury following participation in a disability education session at an allopathic medical school: a qualitative study
Source: Spinal Cord Ser Cases. 2023 Jul 7;9:28. doi: 10.1038/s41394-023-00582-6 (PMC10328920; doi:10.1038/s41394-023-00582-6)
Supplement: Supplementary file 1 — Appendix 1 [file 41394_2023_582_MOESM1_ESM.docx]

**Appendix**

**Focus Group Discussion Guide: Living with SCI/Physical Disabilities**

Researcher #1

**Pre-Discussion Orientation**

*Facilitators introduce themselves and the session. Remind the respondents that at the end of the discussion, they will be asked to give feedback about any of the questions they consider difficult or unclear. They will also be asked about whether there is any unclear language in the description of the individuals with spinal cord injury (SCI) or the training instructions and if they have suggestions for making it more understandable.*

#### I. Self-Introductions and Warm-up

1. What is a focus group discussion?
2. How do focus group discussions work?

- We are interested in your viewpoint, which is critical to our success in understanding your perspectives in helping to teach this class
- focus group is to gather your opinions and perspectives
- no right or wrong answers
- honest answers; don’t be afraid to hurt our feelings if you don’t like something- we would rather get your feedback now than develop an intervention that’s not as good as it can be.
- If a particular question or questions make you uncomfortable, you don’t have to provide an answer – just ask me to move on to the next question.

1. Group defines/outlines rules for mutual respect

- One person talk at a time
- Put cell phones on vibrate
- No judgment

1. Microphones, recording, assurance of privacy

Your input and comments will help us to understand how to best design and deliver this program to improve the health, function and quality of life of people with SCI.

Remember today that you are the expert and we are the students!

***Do you have any questions about the study or the interview before we started?***

***May we turn on the audio recorder now?***

**II. INTRODUCTION** (NOTE: Text is written using lay language)

ICEBREAKER – self-introductions

**Moderator:** describe the goals

- *Today, we are going to discuss your thoughts and get your ideas on how to best to approach students to improve communication about the health, function, and quality of life of patients with SCI*

*Just a quick reminder- There are* ***three*** *ways that you can contribute to today’s discussion:*

1. You can share your personal experience (again, we remind you that what is said here today will remain confidential and anything you tell me won’t be linked back to you).
2. You can also share your thoughts and ideas as they pertain to other persons with SCI or other disabilities. For instance, you can also talk about someone that you know or that you have heard about with similar situation
3. You can share your thoughts about any adjustments that need to be when you (individuals) visit your physician so that it is most appropriate and relevant for use by persons in this community.

***Researcher #2***

- How did the interaction with medical students go?
  - Were you surprised with how much students knew? Didn’t know? (regarding their experiences with people with physical challenges).
- What was the experience like?
  - What was easy for you? Difficult for you? Give us an example.
  - Were students able to grasp your challenges?
  - How did they respond?
    - - Did they have new approaches, ideas, or were they surprised?
  - Did you discuss challenges that you experienced with previous medical providers?
    - What do you think the students learned from the experience?
  - Were there things that were not discussed that you think would be important to incorporate next time?

***Researcher #1***

***Individual’s Experiences with Physicians/Medical Students:***

- When you were asked to describe your worst encounter with a physician. What did you share?
- When you were asked to describe your best encounter with a physician. What did you share?
- During the H&P role play, how prepared were students in terms of:
  - - Did they ask you about your SCI/physical disability?
    - Can you tell me more about your condition/disability/physical challenge?
    - How did it happen?
    - Did the student talk to you or your caregiver?
    - How did you feel during the H&P?
    - Were the students knowledgeable enough to ask about Activities of Daily Living (ADLs) or Instrumental Activities of Daily Living (IADLs)

(toileting, bathing, dressing/grooming/hygiene, eating/feeding, transferring (e.g. from bed to chair), getting from one place to another or preparing food and medications, housekeeping, shopping, managing money, using the telephone/technology/transportation)

***Researcher #2***

- What did you think was most surprising that the students did not know? Please give us an example, basic things
- Did the students ask about what type of assistive devices can better help you (individuals) in your daily lives?
- Did the students ask you what your goals are for the visit? Overall health goals?
- Did they ask about rehab? Did come up during discussions?
- Did the students ask if you exercise? Or what kind of physical activity you do?
- Overall, how was your experience?
- Any final comments or thoughts on things we should know

***IV: Conclusion***

- Thank the individuals for their time and sharing their story.
- Ask them to share one thing they learned from the session.
